# Fusarium spp.: infections and intoxications

**DOI:** 10.3205/id000089

**Published:** 2024-09-27

**Authors:** Herbert Hof, Jens Schrecker

**Affiliations:** 1 Labor Limbach and colleagues, Heidelberg, Germany; 2 Department of Ophthalmology, Rudolf Virchow Klinikum Glauchau, Germany

## Abstract

The genus *Fusarium*, member of the *Hypocreaceae* family, comprises over 500 spp. with an ever-evolving taxonomy. These fungi, some highly pathogenic, primarily affect various plants, including major crops like maize, rice, cereals, and potatoes, leading to significant agricultural losses and contributing to human undernutrition in certain regions. Additionally, *Fusarium* spp. produce harmful mycotoxins like trichothecenes, fumonisins, zearalenones, etc., posing health risks to animals and humans. These toxins generally transferred to food items can cause diverse issues, including organ failure, cancer, and hormonal disturbances, with effects sometimes appearing years after exposure. The fungi’s vast genetic repertoire enables them to produce a range of virulence factors, leading to infections in both animals and humans, particularly in immunocompromised individuals. *Fusarium* spp. can cause systemic infections and local infections like keratitis. Due to limited antifungal effectiveness and biofilm formation, these infections are often challenging to treat with poor outcomes.

## Introduction

*Fusarium* spp. belong to a heterogeneous group of ascomycetous hyalohyphomycetes. This genus comprises a large number of >500 recognized phylogenetic species. Hence, the identification and classification of single strains is challenging and in laboratory routine occasionally frustrating. Molecular methods have been applied in modern times to identify and characterize the various *Fusarium* spp., since the descriptions of micromorphological characters have turned out to be imprecise. Several genes, for example the TEF1α gene, or whole DNA regions, respectively, have been employed to characterize the species boundaries within the genus *Fusarium* [1]. One has to admit, however, that the precise identification is tricky, since the genomic analysis has clustered in species which are morphological quasi identical (cryptic species). The delineation of certain species is indeed sometimes delicate, so that in practice one has created so-called complexes of several strains more or less related to each other. For example, the *F. fujikuroi* complex (teleomorph: *Giberrella* *fujikuroi* complex) consists of about 50 species whereby *F. fujikuroi* (*sensu stricto*), *F. proliferatum* and *F. verticillioides* are the most important ones [2], [3], [4]. The *F. solani* complex (*Nectria solani* complex) is composed of >40 different species [5]. The *F. graminearum* complex (*Gibberella graminearum* complex) yields at least 15 species [1], which differ in their geographic distribution as well as their host preferences. One has to keep in mind that it is hard to recognize in publications whether the reported properties refer to single strains or to entire complexes. *Fusarium* spp. ranged among several distinct teleomorph genera such as *Neocosmospora* [6], *Albonectria*, *Cyanonectria*, *Gibberella*, *Haematonectria* and *Nectria*. The most relevant species are listed in Table 1 (Tab. 1).

The taxonomy of this fungal group is still controversially discussed among experts; there is still a fundamental debate going on [6]. In addition it is confusing that in the literature a fungus will appear under various names [7]; for example *F. fujikuroi* was named *F. moniliforme* formerly. 

MALDI-TOF turned out to be a reliable method in laboratory routine for differentiation of isolates for practical purposes [2], [8].

*Fusarium* spp. are characterized by well-developed, septated, non-pigmented hyphae with acute-angled bifurcations forming typical macroconidia, so-called sporodochial conidia varying in shape, size, and number from one species to another. The microconidia are so-called aleuriospores, which do not originate from specialized conidiophores but directly from the hyphae. These small, hydrophobic spores are easily distributed by air. Sexual reproduction is rarely observed under routine laboratory conditions [7].

*Fusarium* spp. are cosmopolitan hyphomycetes growing ubiquitously especially in soil, water and on plants, namely on roots as well as on leaves [7]. Because of their versatile biologic properties, they play a notable role in nature [9]. Indeed, the majority of *Fusarium* spp., in particular *F. verticillioides* and *F. graminearum*, are primarily plant pathogens effectuating worldwide immense crop losses. This destruction of plants entails an important medical relevance, namely these fungi are the main reasons for hunger and undernutrition representing major medical problems. Virtually all *Fusarium* spp. are capable to produce a more or less wide range of mycotoxins causing severe medical consequences. Unfortunately, these aspects are definitely ignored and/or underestimated by medical doctors [7]. In practice, tests for mycotoxin levels of human specimen are requested rarely, which indicates that during the medical clarification of unclear symptoms intoxications by mycotoxins are not suspected in most instances. In humans, at least certain *Fusarium* spp. can cause a variety of infections, which are highly dependent upon the portal of entry and the immune status of the host [10].

## Plant pathogens

Because of their comprehensive genetic repertoire, *Fusarium* spp. are rather versatile [9] and can settle and thrive under variable conditions, i.e. on different plants [11], whereby some fungi attack particularly roots whereas others prefer leaves. Anyway, certain species have developed host preferences, so that these fungi are found predominantly on specific plants (Table 1 (Tab. 1)), although they may occasionally also affect other hosts, too. Furthermore, there are geographical and climatic distinctions in their local dominance [1]. They may grow saprophytically, i.e. they may degrade dead, organic materials, but sometimes they may behave parasitically, which means that they attack and damage parts of living plants. Fungi are equipped with a large genome and, therefore, possess a broad array of genes, which can be involved in plant infections. Fungi are real chemical factories in producing enzymes. Virulent strains, for their parts, produce large amounts of secretory proteins and cell-wall-degrading enzymes, which are able to damage the host und to induce diseases [12]. Biofilm formation seems to play a crucial role in the *Fusarium* induced plant diseases [11]. In addition, mycotoxins are accused to play a role in plant disease development, since some are phytotoxic [13]. Conversely, mycotoxins may function as fungal virulence factors in plant infections promoting the expansion in a host [14] (Table 2 (Tab. 2)).

In agriculture *Fusarium* spp. play an immense role, since some fungi may produce devastating pests in the fields. One of the most relevant pests are induced by *F. graminearum* in wheat, barley, oats, rye and triticale, inducing so-called Fusarium head blind (sometimes called Fusarium ear blind); also other *Fusarium* spp., such as *F. tricinctum*, are accused to trigger such plant diseases. *F. verticillioides* is infesting especially maize [7]. *F. fujikuroi*, on the other hand, is responsible for maize ear rot, soybean root rot, and in particular for bakanae in rice [3]. *F. oxysporum* may cause banana wilt (also known as Panama disease) [11]. Furthermore, *F. oxysporum* causes wilt diseases in many popular garden and greenhouse flowers and are most serious and common in aster, chrysanthemum, gladiolus, lily, and narcissus. In mimosa wilt the fungus *F. oxysporum* enters through the roots and spreads into the relatively large xylem vessels. The interruption of the water flow to the leaves will result in wilt disease. Because of their ability to produce large numbers of infective conidia, the fungi are able to spread rapidly by air even over long distances. This propagation will be particularly detrimental in monocultures, where pests often infest vast areas.

Hence, *Fusarium* spp. destroy considerable amounts of crop yields annually, causing a huge loss, and lead to a massive reduction of the economic income in the producer countries on various continents of the world [3].

Therapy often fails, which will be due either to resistance of *Fusarium* spp. to the agents used (i.e. in most cases azoles) [15] or to the fact that they form biofilms [11]. Hence, the prevention of the propagation of fungal conidia is of crucial importance. Therefore, it is a frequent practice to utilize large quantities of synthetic fungicides, i.e. pesticides, for prophylaxis. Large amounts of different azole derivatives are applied in agriculture to minimize the risks of fungal infections of food crops and of toxin production, accepting the risk of emergence of resistant strains arising inevitably after persistent administration [16], [17]. Recently, eco-friendly strategies, such as biocontrol, have become applicable more and more [17].

Quite another aspect is the property of some *Fusarium* spp., such as *F. fujikuroi* [12], to produce gibberellin, which exerts stimulatory effects on the growth of some plants.

## Mycotoxins

By definition mycotoxins are products of the secondary metabolic pathways during late logarithmic growth of moulds [18], [19]. In fact, more than 400 different mycotoxins and their metabolites are described. This heterogeneous group of toxic substances [18], [19] play a role in phytopathology as well as in animal and human pathophysiology [13]. Whereas a few mycotoxins are stored in the fungal conidia and thereupon are distributed by air, the vast majority of mycotoxins, including the *Fusarium* mycotoxins, are sectored into the surroundings and hence contaminate a variety of foodstuffs. Foodstuffs recognized as the most risky for *Fusarium* mycotoxins are maize [13], grains, rice, beans, coffee, wine, fruits, nuts, spices, eggs, and meat products after carryover. The problem is that their occurrence is not fully preventable in spite of research efforts and mitigation strategies. Consequently, preharvest contamination of both foods and feeds with *Fusarium* mycotoxin is an almost inevitable phenomenon worldwide [14], [17], [20], [21]. 

The most important *Fusarium* mycotoxins are the trichothecenes (including deoxynivalenol (also known as vomitoxin), nivalenol and T-2 toxin besides zearalenones and fumonisins (Table 3 (Tab. 3)). All these agents are ingested by food; the consequences for humans are not really known and are largely underestimated [2], [17], [20], [21].

Among the so-called “emerging *Fusarium* mycotoxins” moniliformin, enniatins and beauvericin should be mentioned. Their true role is not yet well established and understood [17], [21].

The numbers of various toxins and the amounts produced are determined by the genetic equipment of the fungal strains. In addition, environmental conditions can be crucial. In a special situation, it is hard to predict the extent of the problem. *Fusarium* mycotoxins occur frequently in many foods but fortunately at low concentrations, so there is a need to provide sensitive and reliable methods for their detection. But they can be accumulated in the tissues of cereals and vegetables in high, i.e. harmful, concentrations. In general, maize and rice can be contaminated in high concentrations [3]. Many toxins like fumonisins and trichothecenes are heat-stable and cannot be deactivated by cooking. The different mycotoxins exert their toxic effects in living creatures by quiet diverse metabolic processes [22]. Acute intoxications are often described in animals fed with highly contaminated feeds but are rather rare in humans – at least in developed countries – but may occur after exposure to excessive doses, especially in situations like war and natural catastrophes. By far the most illnesses are related to chronic or repeated exposure. Since the toxic consequences will manifest a long time after the exposure, the individual will not remember the risk at the time when the health problems are noticed. Hence, it is difficult to recognize that there is a causal link between the former mycotoxin intake and the actual disease symptoms. Obviously, in these disease entities the mycotoxins are a pathogenicity factor but not virulence factors, which means that the producing strain will not profit from its performance [23].

One has to keep in mind that co-contamination with mycotoxins from other molds may also occur in food items and their synergistic activities can augment the health injuries.

The only way to surpass the threat posed by *Fusarium* mycotoxins is to prevent or inhibit the production of mycotoxins in the field [17]. Laboratory survey of mycotoxin pollution of food items is of concern to note risky items that should be eliminated from the food chain [21].

## Animal infections

For example, in aquatic animals such as seahorses and dolphins *Fusarium* spp. are able to cause opportunistic infections. The clinical manifestations include local infections such as keratitis or local skin invasion but also organ infections of lungs, liver, cartilage and so on [24]. In sea turtles, for example, they may attack the eggs when they hatch secured by sand under states of high stickiness and a warm and consistent temperature. They disturb the embryo development which finally is responsible for the dramatic egg mortality leading to a decline of turtle population worldwide [24].

In dogs, horses and cattle keratitis can be induced as well as invasive sinusitis [24].

By the way, the exposure of animals to high concentrations of mycotoxins may lead to leukoencephalomalacia, pulmonary edema or liver injury [24].

## Human infections

From a medical perspective, *Fusarium* spp. are rather harmless environmental microbes rarely causing human infections [25], [26].

### Disseminated infections

This entity represents a threatening situation, since the outcome of these infections occuring in already sick people is generally rather poor, not least due to the facts that exact diagnosis is often established late. The symptoms are in most cases not pathognomonic so that the suspicion of *Fusarium* infection by clinicians emerges delayed, often only in case of refractory antibacterial and antifungal treatment. The awareness of *Fusarium* infections is still modest. Furthermore, the laboratory results including exact, reliable differentiation as well as susceptibility testing are available after tedious processes only. In addition, the susceptibility to antifungal agents is generally low. 

In humans, *Fusarium* spp. cause a variety of infections, which are highly dependent upon the portal of entry and the immune status of the host. Invasion via colonized catheters is a common cause of such manifestations. In severely immunocompromised patients, for example due to leukemia, opportunistic *Fusarium* spp. are able to induce locally restricted cutaneous inflammations. There is, however, a tendency to disseminate usually associated with positive blood cultures. It should be emphasized that *Fusarium* spp. – in contrast to *Aspergillus fumigatus* – are principally capable of adventitious sporulation, which allows positive blood cultures and dissemination of conidia via the blood [27]. Indeed, besides fungemia practically all organs may be affected; the most common manifestations are peritonitis in patients receiving dialysis, thrombophlebitis, arthritis, osteomyelitis, endophthalmitis, sinusitis and pneumonia [10]. Even neurologic infections have been reported [28]. The species that are most commonly involved in human infection are *Fusarium solani*, followed by *Fusarium oxysporum* and *Fusarium verticillioides* (out of the *F. fujikuroi* complex; Table 1 (Tab. 1)) [10].

Nosocomial infections of immunocompromised patients have been reported, whereby water distribution systems (drains, faucet aerators, shower heads, sanitary installations) in hospitals are most likely to be the mechanism of aerial dispersal of the conidia responsible for the transmission to the host. Furthermore, airborne conidia may also represent a relevant source of infection [24].

### Local infections – on focus: keratitis

In immunocompetent people, *Fusarium* spp. may cause superficial infections such as onychomycosis [10], [24], [26], whereby it should be critically assessed in each case whether the ubiquitous *Fusarium* spp. are really the etiologic agents of the disease or only contaminants. Most probably the ability of *Fusarium* spp. to trigger nail infections [26] is overestimated. In principle, *Fusarium* spp. are able to form biofilms on the surface of nails, hampering eradication [24]. 

Keratitis due to *Fusarium* spp. is a relevant entity. It is rather common in certain geographical areas such as India [29], which is due to a strong prevalence of fungal conidia in these areas leading to a higher exposition [5] and to predisposing factors such as an enhanced susceptibility of people, possibly because of concomitant irritations of the eyes by other stimuli. The predisposing factors are numerous but often remain unclear in an individual case. The major risk factors are use of contact lenses and trauma or operative intervention damaging the cornea, or blocked tear ducts [30]. In Germany, where several dozens of cases have been described over the last ten years, the majority of affected patients are otherwise healthy women of approximately 50 years of age [31].

Although fungal keratitis is often associated with trauma and prior application of corticosteroids [32], wearing of contact lenses, especially in combination with inadequate hygiene precautions and mold-growth permissive storage fluids, is an important risk factor for such an infection [33].

Despite meeting sterilization and antimicrobial standards by the lens manufacturer, poor hygiene practices and improper interactions with lenses and storage equipment can lead to contamination by *Fusarium* spp. Drying, dilution, or antimicrobial component absorption by the lenses along with the abilities of *Fusarium* spp. for rapid growth and penetration contribute to the risk. The omission of the manual cleaning step in the solution’s use was also identified as a significant risk factor for developing fungal keratitis [34]. Lens care solutions within contact lens cases can become concentrated and form dried films due to evaporation or because the cases are topped off by the user instead of being emptied, cleaned, and refilled regularly [35]. Zhang et al. [5] showed that such films on plastic surfaces of lens cases can support the growth of selective isolates of fungus.

Storage fluids for contact lenses differ in relation to their fungicidal ability. Common products are based on disinfectant agents like Aldox 0.0006% and polyquaternium 0.001%, H_2_O_2_ 3% or PHMB 0.0001%. Schrenker et al. [36] concluded that the risk of *Fusarium* spp. contaminations may be enhanced by the usage of PHMB-based storage fluids in comparison to formulations based on 3% hydrogen peroxide or Aldox. The effect of PHMB may be enhanced by the addition of pH-regulators, but the effect is variable and difficult to assess in real life use. Schrenker’s data showed that storage fluids containing either 3% hydrogen peroxide or Aldox are highly effective against *Fusarium* spp. and prevent contamination of contact lenses with fungal conidia [36].

Another possible factor for the increased incidence of *Fusarium* keratitis among contact lens wearers may be partly due to the formation of biofilms by fusaria on lenses, lens cases, corneal tissue, or a combination of these surfaces [35]. Imamura [35] developed a reproducible *in vitro* model of fungal biofilm formation on contact lenses and demonstrated that *Fusarium* and *Candida* can form biofilms on commonly used soft contact lenses and that the amount, metabolic activity, thickness, and architecture of these fungal biofilms is influenced by the surface properties of the lenses used [35]. Ahearn et al. [37] showed that *Fusarium* mats (in contrast to more tightly bounded candida biofilms) tended to be loosely associated with the lenses and could be released from the lens surface by vigorous shaking or rinsing of the lens. However, there are also findings that the attachment to the lens surface varies from a loose association of conidia and hyphae to firmly attached hyphae that are difficult or impossible to remove [35]. The role of biofilm formation in fungal keratitis still needs to be further investigated.

Occasionally, outbreaks due to contaminated lens solutions are reported [33], [38].

*Fusarium* spp. are equipped with a variety of virulence factors such as mannoproteins on their surface, enabling them to adhere to laminins, fibronectins and collagens on the cornea, where they are able propagate at the given temperature [30]. Furthermore, *Fusarium* spp. are able to create a biofilms on the surface of a cornea – not only on plants [11]. This protects them against defense mechanisms of the innate immunity. Because of their large genetic repertoire *Fusarium* spp. produce in large amounts an array of proteases, phospholipase and cytotoxic peptides, neutralizing antimicrobial oligopeptides, such as lysozyme and defensins, of the humoral, non-specific defences [39].

**Figure 1:**
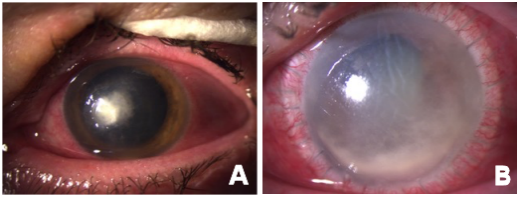
Keratitis in an immunocompetent patient by *F. tonkinense* (see [39]) A: Left eye with a whitish corneal infiltrate upon hospital admission B: The same eye, several weeks after large-diameter repeat keratoplasty: despite temporal stagnation under highly intensive local and systemic therapy, the infection spread within the anterior segment and finally into the vitreous.

Often, a fungal keratitis does not remain limited to the cornea but rather breaks through the anatomical barrier, namely the Descemet’s membrane, (Figure 1 (Fig. 1)) by the help of their virulence factors allowing the pathogen to penetrate the inner eye and to cause endophthalmitis eventually [30], [39], [40]. Such a fatal propagation may finally require an enucleation of the eye [39]. 

Various *Fusarium* spp. are able to cause keratitis but members of the *Fusarium solani* complex such *F. petroliphilum*, *F. keratoplasticum*, *Fusarium tonkinense* and *F. solani* (sensu stricto) are the prevailing agents [31].

## Therapy

Antimicrobial testing of *Fusarium* spp. is not performed routinely, because the standard *in vitro* test methods are not broadly approved. EUCAST (European Committee on Antimicrobial Susceptibility Testing) and CLSI (Clinical and Laboratory Standards Institute) recommendations of performing *in vitro* susceptibility tests differ partially such as inoculum size, glucose composition, pH of the medium, incubation temperature and duration, which may influence the minimum inhibitory concentrations (MICs) values. Furthermore, the interpretation of laboratory results is problematic, since the correlation between *in vitro* susceptibility tests and clinical outcome is sometimes poor. Hence, antifungal susceptibility testing can predict the outcome of treatment only in main traits. Low MICs do not guarantee clinical success, while high MICs are associated with lower probability of a favorable response to a given antifungal agent. In spite of these inconsistencies, *in vitro* testing remains useful in guiding clinicians in taking the right therapeutic decision.

The therapeutic management of *Fusarium* infections, localized or disseminated, is usually challenging due to the site of infection, the underlying disease, and the intrinsic resistance to many antifungal agents currently available [41]. Especially *F. solani* is rather resistant to typical antifungal agents such as azoles and often disposes elevated MICs of amphotericin B. The most effective antifungal in treating* F. solani* infections is amphotericin B, although even this agent has only modest success in the treatment of serious systemic infection [24], [41]. Indeed, all *Fusarium* species are naturally resistant to echinocandins and flucytosine.

The prognosis of disseminated fusarioses is generally rather poor [41] with survival rates at day 90 post diagnosis of 13% to 21%, depending on underlying conditions. Based on the results of clinical studies, the European Society for Microbiology and Infectious Diseases has recommended the use of voriconazole as first-line therapy for invasive *Fusarium* infections regardless of the causative species [42]. Ruhnke et al. [43] suggest the combination of liposomal amphotericin B and voriconazole in severely sick patients and posaconaole as an alternative and in addition surgical removal of infected sites. Newer azole derivatives such as posaconazole [41], [44] and isavuconazole [45] can be considered as an alternative for prophylaxis and salvage therapy, although even these agents may have no reliable activity, because some fungi have undergone mutations, which render azoles generally rather ineffective [15], [24].

According to the generally accepted Tarragona principles [46] for antibiotic therapy of severe infectious diseases, the therapy should start as early as possible; this holds also true for *Fusarium* infections [47].

The therapy of keratitis is also challenging. Besides natamycin, which can be applied only topically, amphotericin B is prefered for the first-line therapy [39]. Natamycin in combination with voriconazole has also been recommended for fusarial keratitis [48]. It could be expected that in the future other azole derivatives such as posaconazole [44] and isavuconazole [45] can be considered as alternatives, although comprehensive ophthalmologic experience with these new azoles is still lacking.

The therapeutic success of antifungals is not only dependent on the *in vitro* activity of agents [31] but can be impaired by the biofilm production by *Fusarium* spp. In case that antifungals (given topically or systematically) fail, surgical interventions, for example a keratoplasty (Figure 1B (Fig. 1)), are indicated [39].

## Notes

### Competing interests

The authors declare that they have no competing interests.

## Figures and Tables

**Table 1:**
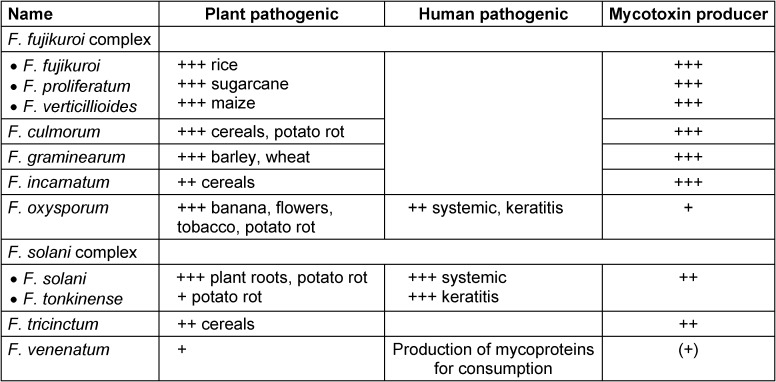
List of some relevant *Fusarium* spp. and their major roles

**Table 2:**
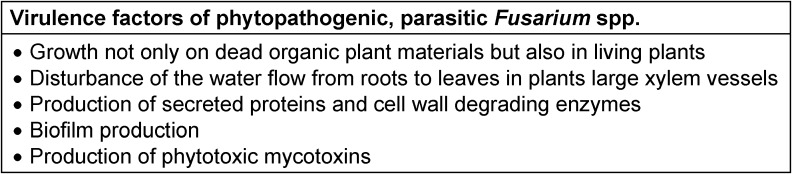
Virulence factors of phytopathogenic, parasitic *Fusarium* spp.

**Table 3:**
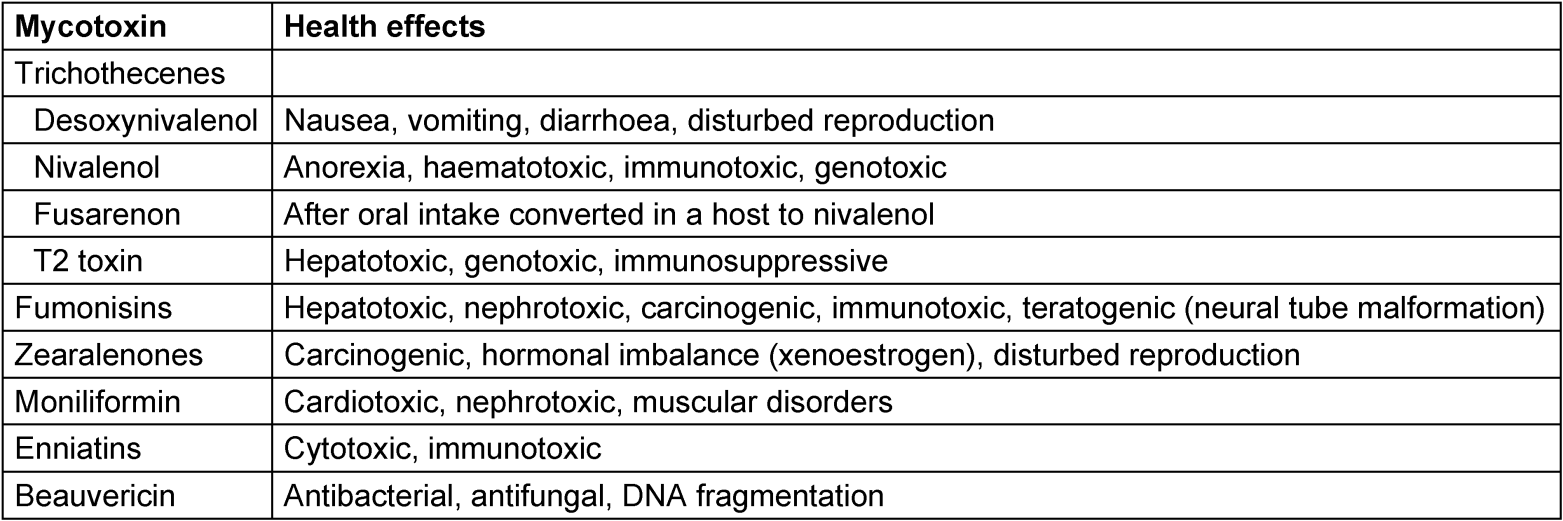
The most relevant *Fusarium* mycotoxins for human health (according to [21])

## References

[1] Hafez M, Gourlie R, Telfer M, Schatz N, Turkington TK, Beres B, Aboukhaddour R (2022). Diversity of Fusarium spp. Associated with Wheat Node and Grain in Representative Sites Across the Western Canadian Prairies. Phytopathology.

[2] Wigmann ÉF, Behr J, Vogel RF, Niessen L (2019). MALDI-TOF MS fingerprinting for identification and differentiation of species within the Fusarium fujikuroi species complex. Appl Microbiol Biotechnol.

[3] Qiu J, Lu Y, He D, Lee YW, Ji F, Xu J, Shi J (2020). Fusarium fujikuroi Species Complex Associated With Rice, Maize, and Soybean From Jiangsu Province, China: Phylogenetic, Pathogenic, and Toxigenic Analysis. Plant Dis.

[4] Noorabadi MT, Masiello M, Taherkhani K, Zare R, Torbati M, Haidukowski M, Somma S, Logrieco AF, Moretti A, Susca A (2021). Phylogeny and mycotoxin profile of Fusarium species isolated from sugarcane in Southern Iran. Microbiol Res.

[5] Zhang N, O'Donnell K, Sutton DA, Nalim FA, Summerbell RC, Padhye AA, Geiser DM (2006). Members of the Fusarium solani species complex that cause infections in both humans and plants are common in the environment. J Clin Microbiol.

[6] Sandoval-Denis M, Lombard L, Crous PW (2019). Back to the roots: a reappraisal of Neocosmospora. Persoonia.

[7] Hof H (2020). The Medical Relevance of Fusarium spp. J Fungi (Basel).

[8] Hendrickx M (2017). MALDI-TOF MS and filamentous fungal identification: a success story?. Curr Fungal Infect Rep.

[9] Tupaki-Sreepurna A, Kindo AJ (2018). Fusarium: The versatile pathogen. Indian J Med Microbiol.

[10] Cighir A, Mare AD, Vultur F, Cighir T, Pop SD, Horvath K, Man A (2023). Fusarium spp. in Human Disease: Exploring the Boundaries between Commensalism and Pathogenesis. Life (Basel).

[11] Ismaila AA, Ahmad K, Siddique Y, Wahab MAA, Kutawa AB, Abdullahi A, Zobir SAM, Abdu A, Abdullah SNA (2023). Fusarium wilt of banana: current update and sustainable disease control using classical and essential oils approaches. Horticultural Plant Journal.

[12] Tadasanahaller PS, Bashyal BM, Yadav J, Krishnan Subbaiyan G, Ellur RK, Aggarwal R (2023). Identification and Characterization of Fusarium fujikuroi Pathotypes Responsible for an Emerging Bakanae Disease of Rice in India. Plants (Basel).

[13] Bryła M, Pierzgalski A, Zapaśnik A, Uwineza PA, Ksieniewicz-Woźniak E, Modrzewska M, Waśkiewicz A (2022). Recent Research on Fusarium Mycotoxins in Maize-A Review. Foods.

[14] Perincherry L, Lalak-Kańczugowska J, Stępień Ł (2019). Fusarium-Produced Mycotoxins in Plant-Pathogen Interactions. Toxins (Basel).

[15] Vermeulen P, Gruez A, Babin AL, Frippiat JP, Machouart M, Debourgogne A (2022). CYP51 Mutations in the Fusarium solani Species Complex: First Clue to Understand the Low Susceptibility to Azoles of the Genus Fusarium. J Fungi (Basel).

[16] Hof H (2001). Critical annotations to the use of azole antifungals for plant protection. Antimicrob Agents Chemother.

[17] Xue H, Liu Q, Yang Z (2023). Pathogenicity, Mycotoxin Production, and Control of Potato Dry Rot Caused by Fusarium spp.: A Review. J Fungi (Basel).

[18] Bennett JW, Klich M (2003). Mycotoxins. Clin Microbiol Rev.

[19] Keller NP (2019). Fungal secondary metabolism: regulation, function and drug discovery. Nat Rev Microbiol.

[20] Munkvold GP (2017). Fusarium Species and Their Associated Mycotoxins. Methods Mol Biol.

[21] Ekwomadu TI, Akinola SA, Mwanza M (2021). Fusarium Mycotoxins, Their Metabolites (Free, Emerging, and Masked), Food Safety Concerns, and Health Impacts. Int J Environ Res Public Health.

[22] Awuchi CG, Ondari EN, Nwozo S, Odongo GA, Eseoghene IJ, Twinomuhwezi H, Ogbonna CU, Upadhyay AK, Adeleye AO, Okpala COR (2022). Mycotoxins' Toxicological Mechanisms Involving Humans, Livestock and Their Associated Health Concerns: A Review. Toxins (Basel).

[23] Hof H (2008). Mycotoxins: pathogenicity factors or virulence factors?. Mycoses.

[24] Sáenz V, Alvarez-Moreno C, Pape PL, Restrepo S, Guarro J, Ramírez AMC (2020). A One Health Perspective to Recognize Fusarium as Important in Clinical Practice. J Fungi (Basel).

[25] Nucci F, Nouér SA, Capone D, Nucci M (2018). Invasive mould disease in haematologic patients: comparison between fusariosis and aspergillosis. Clin Microbiol Infect.

[26] Thomas B, Audonneau NC, Machouart M, Debourgogne A (2020). Fusarium infections: Epidemiological aspects over 10 years in a university hospital in France. J Infect Public Health.

[27] Lockwood MB, Crescencio JC (2016). Adventitious sporulation in Fusarium: The yeast that were not. IDCases.

[28] Góralska K, Blaszkowska J, Dzikowiec M (2018). Neuroinfections caused by fungi. Infection.

[29] Ghosh AK, Gupta A, Rudramurthy SM, Paul S, Hallur VK, Chakrabarti A (2016). Fungal Keratitis in North India: Spectrum of Agents, Risk Factors and Treatment. Mycopathologia.

[30] Niu L, Liu X, Ma Z, Yin Y, Sun L, Yang L, Zheng Y (2020). Fungal keratitis: Pathogenesis, diagnosis and prevention. Microb Pathog.

[31] Walther G, Zimmermann A, Theuersbacher J, Kaerger K, von Lilienfeld-Toal M, Roth M, Kampik D, Geerling G, Kurzai O (2021). Eye Infections Caused by Filamentous Fungi: Spectrum and Antifungal Susceptibility of the Prevailing Agents in Germany. J Fungi (Basel).

[32] Alfonso EC, Cantu-Dibildox J, Munir WM, Miller D, O'Brien TP, Karp CL, Yoo SH, Forster RK, Culbertson WW, Donaldson K, Rodila J, Lee Y (2006). Insurgence of Fusarium keratitis associated with contact lens wear. Arch Ophthalmol.

[33] Chang DC, Grant GB, O'Donnell K, Wannemuehler KA, Noble-Wang J, Rao CY, Jacobson LM, Crowell CS, Sneed RS, Lewis FM, Schaffzin JK, Kainer MA, Genese CA, Alfonso EC, Jones DB, Srinivasan A, Fridkin SK, Park BJ, Fusarium Keratitis Investigation Team (2006). Multistate outbreak of Fusarium keratitis associated with use of a contact lens solution. JAMA.

[34] Ahearn DG, Zhang S, Stulting RD, Schwam BL, Simmons RB, Ward MA, Pierce GE, Crow SA (2008). Fusarium keratitis and contact lens wear: facts and speculations. Med Mycol.

[35] Imamura Y, Chandra J, Mukherjee PK, Lattif AA, Szczotka-Flynn LB, Pearlman E, Lass JH, O'Donnell K, Ghannoum MA (2008). Fusarium and Candida albicans biofilms on soft contact lenses: model development, influence of lens type, and susceptibility to lens care solutions. Antimicrob Agents Chemother.

[36] Schrenker B, Zimmermann A, Koch T, Walther G, Martin R, Kampik D, Kurzai O, Theuersbacher J (2024). Polyhexanide based contact lens storage fluids frequently exhibit insufficient antifungal activity against Fusarium species. Int J Med Microbiol.

[37] Ahearn DG, Simmons RB, Zhang S, Stulting RD, Crow SA, Schwam BL, Pierce GE (2007). Attachment to and penetration of conventional and silicone hydrogel contact lenses by Fusarium solani and Ulocladium sp. in vitro. Cornea.

[38] Khor WB, Aung T, Saw SM, Wong TY, Tambyah PA, Tan AL, Beuerman R, Lim L, Chan WK, Heng WJ, Lim J, Loh RS, Lee SB, Tan DT (2006). An outbreak of Fusarium keratitis associated with contact lens wear in Singapore. JAMA.

[39] Schrecker J, Seitz B, Berger T, Daas L, Behrens-Baumann W, Auw-Hädrich C, Schütt S, Kerl S, Rentner-Andres S, Hof H (2021). Malignant Keratitis Caused by a Highly-Resistant Strain of Fusarium Tonkinense from the Fusarium Solani Complex. J Fungi (Basel).

[40] Mahmoudi S, Masoomi A, Ahmadikia K, Tabatabaei SA, Soleimani M, Rezaie S, Ghahvechian H, Banafsheafshan A (2018). Fungal keratitis: An overview of clinical and laboratory aspects. Mycoses.

[41] Hoenigl M, Salmanton-García J, Walsh TJ, Nucci M, Neoh CF, Jenks JD, Lackner M, Sprute R, Al-Hatmi AMS, Bassetti M, Carlesse F, Freiberger T, Koehler P, Lehrnbecher T, Kumar A, Prattes J, Richardson M, Revankar S, Slavin MA, Stemler J, Spiess B, Taj-Aldeen SJ, Warris A, Woo PCY, Young JH, Albus K, Arenz D, Arsic-Arsenijevic V, Bouchara JP, Chinniah TR, Chowdhary A, de Hoog GS, Dimopoulos G, Duarte RF, Hamal P, Meis JF, Mfinanga S, Queiroz-Telles F, Patterson TF, Rahav G, Rogers TR, Rotstein C, Wahyuningsih R, Seidel D, Cornely OA (2021). Global guideline for the diagnosis and management of rare mould infections: an initiative of the European Confederation of Medical Mycology in cooperation with the International Society for Human and Animal Mycology and the American Society for Microbiology. Lancet Infect Dis.

[42] Tortorano AM, Richardson M, Roilides E, van Diepeningen A, Caira M, Munoz P, Johnson E, Meletiadis J, Pana ZD, Lackner M, Verweij P, Freiberger T, Cornely OA, Arikan-Akdagli S, Dannaoui E, Groll AH, Lagrou K, Chakrabarti A, Lanternier F, Pagano L, Skiada A, Akova M, Arendrup MC, Boekhout T, Chowdhary A, Cuenca-Estrella M, Guinea J, Guarro J, de Hoog S, Hope W, Kathuria S, Lortholary O, Meis JF, Ullmann AJ, Petrikkos G, Lass-Flörl C, European Society of Clinical Microbiology and Infectious Diseases Fungal Infection Study Group, European Confederation of Medical Mycology (2014). ESCMID and ECMM joint guidelines on diagnosis and management of hyalohyphomycosis: Fusarium spp., Scedosporium spp. and others. Clin Microbiol Infect.

[43] Ruhnke M, Cornely OA, Schmidt-Hieber M, Alakel N, Boell B, Buchheidt D, Christopeit M, Hasenkamp J, Heinz WJ, Hentrich M, Karthaus M, Koldehoff M, Maschmeyer G, Panse J, Penack O, Schleicher J, Teschner D, Ullmann AJ, Vehreschild M, von Lilienfeld-Toal M, Weissinger F, Schwartz S (2020). Treatment of invasive fungal diseases in cancer patients-Revised 2019 Recommendations of the Infectious Diseases Working Party (AGIHO) of the German Society of Hematology and Oncology (DGHO). Mycoses.

[44] Wiederhold NP, Najvar LK, Bocanegra R, Graybill JR, Patterson TF (2010). Efficacy of posaconazole as treatment and prophylaxis against Fusarium solani. Antimicrob Agents Chemother.

[45] Broutin A, Bigot J, Senghor Y, Moreno-Sabater A, Guitard J, Hennequin C (2020). In Vitro Susceptibility of Fusarium to Isavuconazole. Antimicrob Agents Chemother.

[46] Sandiumenge A, Diaz E, Bodí M, Rello J (2003). Therapy of ventilator-associated pneumonia. A patient-based approach based on the ten rules of “The Tarragona Strategy”. Intensive Care Med.

[47] Guerrero Arias CA, Marulanda Nieto CJ, Díaz Gómez CJ (2022). Fusarium spp. infection: The importance of an early diagnosis. Enferm Infecc Microbiol Clin (Engl Ed).

[48] Al-Hatmi AMS, Bonifaz A, Ranque S, Sybren de Hoog G, Verweij PE, Meis JF (2018). Current antifungal treatment of fusariosis. Int J Antimicrob Agents.

